# Deacetylation of HSC70 by SIRT2 promotes chaperone mediated autophagy

**DOI:** 10.1080/27694127.2025.2580781

**Published:** 2025-11-12

**Authors:** Byunghyun Ahn, Wenzhe Chen, Wenbiao Shi, Ruben Shrestha, Fenghua Hu, Hening Lin

**Affiliations:** a Department of Chemistry and Chemical Biology, Cornell University, Ithaca, NY, USA; b Department of Molecular Biology and Genetics, Cornell University, Ithaca, NY, USA; c Weill Institute for Cell and Molecular Biology, Cornell University, Ithaca, NY, USA; d Howard Hughes Medical Institute, Department of Medicine and Department of Chemistry, University of Chicago, Chicago, IL, USA; e Department of Nutrition and Health, China Agricultural University, Beijing, China; f Key Laboratory of Precision Nutrition and Food Quality, Beijing, China; g Bruker Daltonics, San Jose, CA, USA; h Howard Hughes Medical Institute, Cornell University, Ithaca, NY, USA

**Keywords:** Chaperone-mediated autophagy, lysosomes, heat shock chaperones, sirtuin, protein degradation, deacetylation, SIRT2, HSC70, KFERQ motif, amino acids starvation

## Abstract

Chaperone-mediated autophagy (CMA) is a selective form of lysosomal protein degradation essential for cellular proteostasis. CMA is activated during cellular stress, such as starvation, and involves the chaperone protein HSC70 (HSPA8) recognizing substrates containing KFERQ-like motifs. However, the regulatory mechanisms governing CMA activation remain poorly understood. Here, we demonstrate that the NAD^+^ -dependent deacetylase SIRT2 promotes CMA activation by deacetylating HSC70 at lysine 557 (K557). Our findings reveal that SIRT2 activity is upregulated during starvation, enhancing its interaction with HSC70 and facilitating the deacetylation of K557. Deacetylation of HSC70 at K557 increases its binding affinity to CMA substrates, thereby promoting their lysosomal degradation. Mutation of K557 to a deacetylation-mimetic arginine (K557R) enhances CMA activity under both nutrient-rich and starvation conditions, while the acetylation-mimetic glutamine mutant (K557Q) impairs substrate binding and CMA activation. Furthermore, the inhibition or knockdown of SIRT2 reduces CMA activity, which is rescued by HSC70 K557R expression. These findings identify SIRT2-mediated deacetylation of HSC70 as a regulatory mechanism for CMA activation during nutrient deprivation and highlight the role of protein lysine acetylation in proteostasis. This study provides insights into the interplay between SIRT2, HSC70, and CMA, with potential implications for diseases linked to proteostasis dysregulation, including neurodegenerative disorders and cancer.

## Introduction

The heat shock family of proteins (HSP) plays a crucial role in maintaining cellular proteostasis (protein homeostasis) through proper protein folding of nascent polypeptides [1], degradation of misfolded and unfolded proteins [2], prevention of protein aggregation [3], and disaggregation of protein aggregates [4], especially in cellular stress conditions such as heat, cold, nutrient starvation, oxidative stress and other environmental stress [5]. The HSP family is highly conserved across species and includes several subfamilies such as HSP70, HSP90, and small HSPs, and each subfamily has a distinct function and mechanisms in different parts of proteostasis. Among these subfamilies, HSP70 proteins are the most notable for their high expression and wide-ranging participation and involvement in cellular proteostasis pathways. Heat shock cognate 71kDa protein (HSC70, gene name HSPA8) is a member of HSP70 subfamily of proteins that is ubiquitously expressed and plays a crucial role as a chaperone in maintaining proteostasis [6–9]. HSC70 is unique in that it is the only HSP protein known to directly binds to substrate proteins for chaperone-mediated autophagy (CMA) [10].

CMA is a selective form of autophagy where specific proteins are targeted for degradation in lysosomes. Substrate proteins of CMA have the target motif of amino acids sequence KFERQ [11], which is recognized by HSC70. The HSC70 chaperone, together with other cochaperones such as carboxyl terminus of HSC70-interacting protein (CHIP), heat shock protein 40 (HSP40 or DNABJ1) and HSP70-HSP90 organizing protein (HOP), binds to the target protein and translocate to the lysosome [12,13], where it interacts with the lysosomal membrane protein LAMP2A (lysosome-associated protein type 2A). The target protein is then unfolded and directed across the lysosomal membrane into the lysosomes, where the target proteins are degraded by lysosomal enzymes. CMA plays a crucial role in maintaining cellular homeostasis by selectively degrading damaged or misfolded proteins, thus preventing accumulation and potential toxicity [14,15]. CMA has been implicated in the pathogenesis of several neurodegenerative diseases and cancer [14]. Importantly, previous studies showed that CMA is activated during starvation and that starvation-induced CMA provides cells with free amino acids for energy generation and synthesis of crucial proteins during the stress conditions [16,17]. Despite the important role of CMA during starvation, the activation signals for CMA remain mostly unknown.

Protein post-translational modifications (PTMs) play a crucial role in cellular processes such as gene expression, protein stability, cellular metabolism, cell cycle, apoptosis, and stress response [18–20]. Recent studies have shown that HSP70 family proteins are highly modified at the post-translational level, implicating the importance of PTMs on the function of the HSP70 family of proteins [21]. More specifically, the HSP70 family of proteins is highly acetylated [21]. For example, a previous study in yeast revealed that Ssa1, a yeast protein belonging to the HSP70 family, is acetylated at four different lysine residues, deacetylation of which is required to interact with crucial co-chaperone proteins during heat shock [22]. Also, HSP70 was shown to be acetylated at K77 as an early response to oxidative stress to participate in protein folding, and deacetylated at K77 during later phases of oxidative stress to promote degradation of damaged proteins [23]. Despite these discoveries, the role of acetylation in HSC70 had been mostly unexplored, although a total of 50 acetylation sites have been identified in HSC70 [21].

Here, we show that starvation-induced deacetylation on HSC70 regulates the binding of HSC70 to CMA substrates with KFERQ-like motif. Mechanistically, Sirtuin 2 (SIRT2), a member of the NAD^+^-dependent protein lysine deacylase family, promote the activation of CMA under nutrient deprivation conditions by deacetylation of HSC70 at K557. Deacetylation of HSC70 at K557 enhances the binding of HSC70 to target protein substrate with KFERQ-like motif, leading to increased CMA activity. Our results provide important mechanistic insights into how CMA is regulated, how HSC70 recognizes CMA substrates, and how SIRT2 regulates proteostasis through CMA.

## Results

### SIRT2 is important for chaperone-mediated autophagy (CMA)

Sirtuin 2 (SIRT2) is a member of the sirtuin family of protein lysine deacylases that can remove both the acetyl group and long-chain fatty acyl groups [24,25]. Interestingly, SIRT2’s activity is upregulated during starvation and SIRT2 has been shown to enhance protein degradation via activation of macroautophagy [26]. CMA is also upregulated during starvation [27]. Therefore, we questioned whether SIRT2 also plays a role in the activation of CMA.

**Figure 1.**
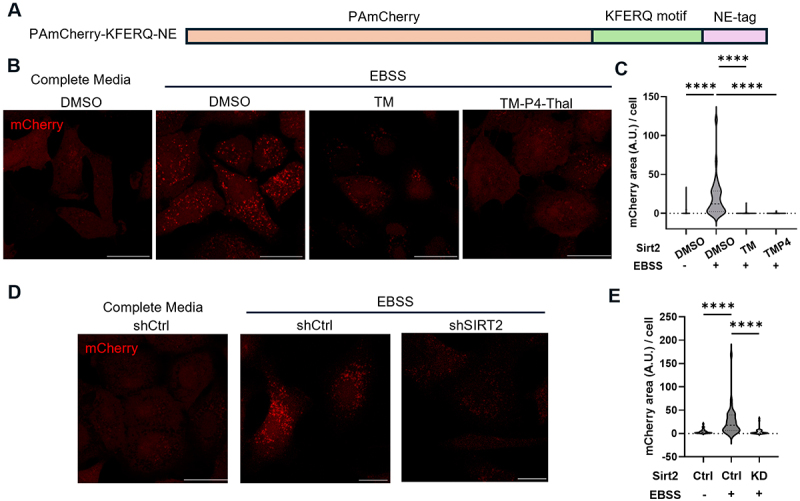
SIRT2 is important for chaperone-mediated autophagy. (A) Plasmid map illustration of PAmCherry-KFERQ-NE. (B) A549 wt cells stably overexpressing PAmCherry-KFERQ-NE under nutrient-rich or starvation conditions and treated with DMSO control, TM, or TM-P4-Thal (TMP4) and observed under fluorescent confocal microscope. (C) Quantification of the data from (B). (D) A549 shCtrl or shSIRT2 cells stably overexpressing PAmCherry-KFERQ-NE under nutrient rich or amino acids starvation conditions were observed as in (B). (E) Quantification of the data from (D). Data are means ± SD. **p < 0.01. Scale bar = 20 μm.

To assess cellular CMA activity, we employed the CMA reporter system KFERQ-PAmCherry, developed by Ho and coworkers [28]. This system utilizes a photoactivatable mCherry (PAmCherry) fused to a sequence derived from Ribonuclease A, which contains the “KFERQ” motif (Figure 1A). Under conditions of increased CMA activity, such as during starvation, the mCherry signal colocalizes with lysosomes, resulting in the appearance of punctate structures. Conversely, under conditions of reduced CMA activity, such as in nutrient-rich environments, the mCherry signal remains dispersed throughout the cytoplasm, with no lysosomal colocalization [28].

We first generated a stable A549 cell line expressing the KFERQ-PAmCherry CMA reporter (Figure S1). As expected, starvation (4 hours of EBSS treatment) led to a significant increase in CMA activity, consistent with previous findings demonstrating starvation-induced CMA (Figure 1B,D) [28]. To investigate the role of SIRT2 in CMA, we assessed CMA activity under conditions of SIRT2 knockdown or inhibition. SIRT2 knockdown was performed using shRNA lentiviral transduction followed by puromycin selection, and SIRT2 inhibition was done by treating the cells with 25 μM of thiomyristoyl (TM) and 2.5 μM of TM-P4-Thalidomide (TM-P4-Thal) for 24 hours. TM is a mechanism-based small-molecule inhibitor of SIRT2 and TM-P4-Thal is a TM-derived PROTAC degrader that degrades SIRT2 protein via the proteasomes [29,30]. We observed a marked decrease in CMA activity following SIRT2 inhibition (Figure 1B,C) or knockdown (Figure 1D,E) under starvation, indicating that SIRT2 promotes CMA activity under starvation conditions.

### HSC70 is deacetylated by SIRT2 under starvation

As a NAD^+^-dependent protein lysine deacylase, the function of SIRT2 in regulating CMA should be achieved through substrate deacylation. We thus sought to identify the substrate protein(s) that could mediate the effect of SIRT2 on CMA. CMA is known to require a chaperone protein, HSC70 (gene name HSPA8). During CMA, HSC70 and its cochaperones recognize and bind to the “KFERQ” motif in the substrate, which is subsequently translocated into the lysosome for degradation via the lysosomal membrane receptor LAMP2A [14,31]. To date, HSC70 remains the only known chaperone protein directly involved in CMA. Given HSC70’s and SIRT2’s involvements in CMA, we hypothesized that SIRT2 May deacetylate HSC70 to regulate CMA. Therefore, we first aimed to determine whether HSC70 is a substrate of SIRT2.

**Figure 2.**
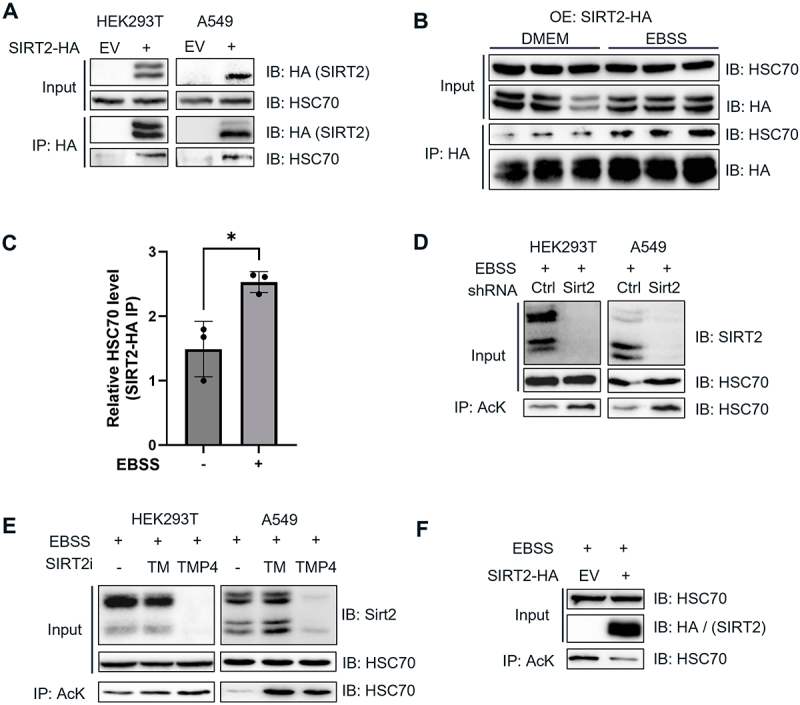
SIRT2 deacetylates HSC70 under starvation. (A) HEK293T and A549 cells were transfected with SIRT2-HA and the interaction of SIRT2-HA and endogenous HSC70 was assessed by immunoprecipitation (IP) and Western blot. (B) HEK293T cells were transfected with SIRT2-HA the interaction of SIRT2-HA and endogenous HSC70 during nutrient-rich conditions (DMEM) and amino acids starvation conditions (EBSS 4 h) was compared using IP and Western blot. (C) Relative HSC70 level pulled down with SIRT2 in (C) was quantified. (D) The acetylation levels of HSC70 in shCtrl and shSIRT2 were measured under starvation conditions using acetyl lysine affinity beads. (E) the acetylation levels of HSC70 in HEK293T cells treated with DMSO control, TM, and TM-P4-Thal (TMP4) were analyzed as in (D). (F) The acetylation levels of HSC70 in HEK293T cells was decreased by SIRT2 overexpression.

We overexpressed HA-tagged SIRT2 in HEK293T cells, performed immunoprecipitation using HA-affinity beads, and detected co-immunoprecipitated HSC70 protein with SIRT2 (Figure 2A), suggesting that HSC70 interacts with SIRT2. Previous studies on SIRT2 demonstrated that the deacetylase activity of SIRT2 is enhanced under starvation conditions, such as those induced by Earle’s Balanced Salt Solution (EBSS) treatment [26,32]. To investigate whether starvation influences the interaction between HSC70 and SIRT2, we treated HEK293T cells with EBSS for 4 hours. Under this starvation conditions, we observed a significant increase in the interaction between HSC70 and SIRT2 (Figure 2B,C), indicating that the HSC70-SIRT2 interaction is increased during starvation.

We next examined whether SIRT2 deacetylates HSC70 under nutrient-deprived conditions. We generated a stable SIRT2-knockdown HEK293T cell line using shRNA lentiviral transduction and found that the acetylation levels of HSC70 were significantly elevated in SIRT2-knockdown cells compared to control cells during starvation (Figure 2D), suggesting that SIRT2 mediates the deacetylation of HSC70. Furthermore, treatment of SIRT2 inhibitor thiomyristoyl (TM) [29] and SIRT2 degrader TM-P4-Thal [30] during starvation conditions increased the acetylation level of HSC70 (Figure 2E). Consistently, brain tissues from SIRT2-CRISPR knockout (SIRT2^− /−^) mice displayed higher HSC70 acetylation levels compared to those from C57BL/6 wild-type (B6WT) mice (Figure S2). Furthermore, SIRT2 overexpression decreases the acetylation level of HSC70 (Figure 2F). Collectively, these findings suggest that SIRT2 deacetylates HSC70 during starvation.

### SIRT2 is essential for HSC70’s CMA substrate binding

Given that SIRT2 deacetylates HSC70 and HSC70 is the only chaperone protein known to directly bind to CMA substrates with KFERQ-like motif, we investigated whether HSC70 deacetylation influences CMA activity. HSC70 contains an N-terminal nucleotide-binding domain (NBD) and a C-terminal substrate-binding domain (SBD), and we observed that 31 of the 48 potential acetylation sites are located within the SBD [33]. This prompted us to explore whether SIRT2 regulates the binding of HSC70 to CMA substrates.

**Figure 3.**
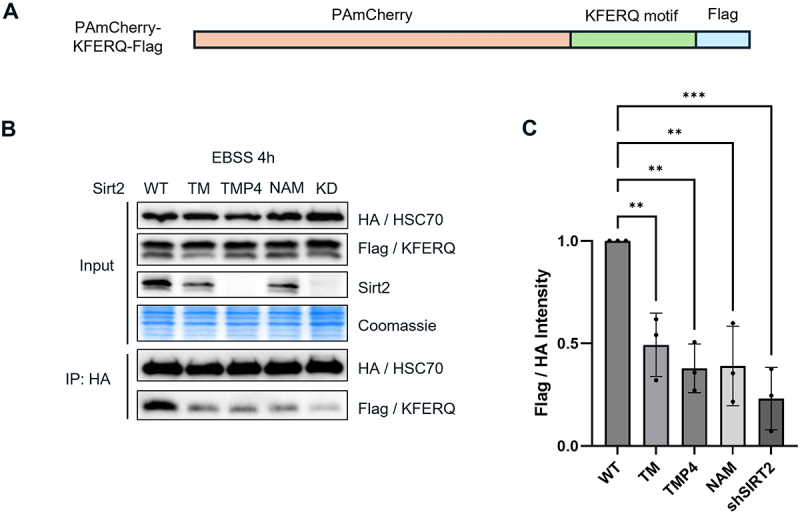
SIRT2 is important for HSC70 binding to CMA substrate. (A) PAmCherry-KFERQ-Flag construct made. (B) Co-IP of HSC70 and mCherry-KFERQ-Flag. HEK293T cells were transfected with HSC70-HA and mCherry-KFERQ-Flag, then starved and treated with SIRT2 inhibitors or SIRT2 knockdown (KD). HSC70 binding to CMA substrates was observed using co-IP and Western blot. (C) Quantification of data from (B). The intensity of Flag signal was normalized with the HA signal, and then the relative intensity of Flag/HA in each lane was calculated and normalized with control (lane 1). shSIRT2 represents SIRT2 KD. Data are means ± SD (*n* = 3). **p < 0.01.

To test this, we constructed a plasmid containing KFERQ-PAmCherry-Flag (Figure 3A) by replacing the NE tag in the plasmid in Figure 1A with Flag tag. We co-overexpressed KFERQ-PAmCherry-Flag and HSC70-HA, and checked for the interaction of HSC70 and its CMA substrate KFERQ using co-immunoprecipitation (co-IP). Our results showed that HSC70 binding to CMA substrates was significantly reduced when SIRT2 was knocked down or inhibited with TM or TM-P4-Thal, or pan-sirtuin inhibitor nicotinamide (NAM) (Figure 3B,C), indicating that SIRT2 is required for optimal interaction between HSC70 and CMA-substrate with KFERQ motif. To confirm that the interaction is via the “KFERQ” motif, we deleted the “KFERQ” motif in the KFERQ-PAmCherry reporter system, which dramatically decreased HSC70’s interaction with the substrate, confirming the important role of the “KFERQ” motif in CMA activity (Figure S3). Furthermore, we picked two known endogenous CMA substrates, IκBα and GAPDH, and showed that HSC70 binding to them was significantly reduced when SIRT2 was inhibited with TM or TM-P4-Thal (Figure S4).

### SIRT2 deacetylation at K557 regulates HSC70’s CMA substrate binding

**Figure 4.**
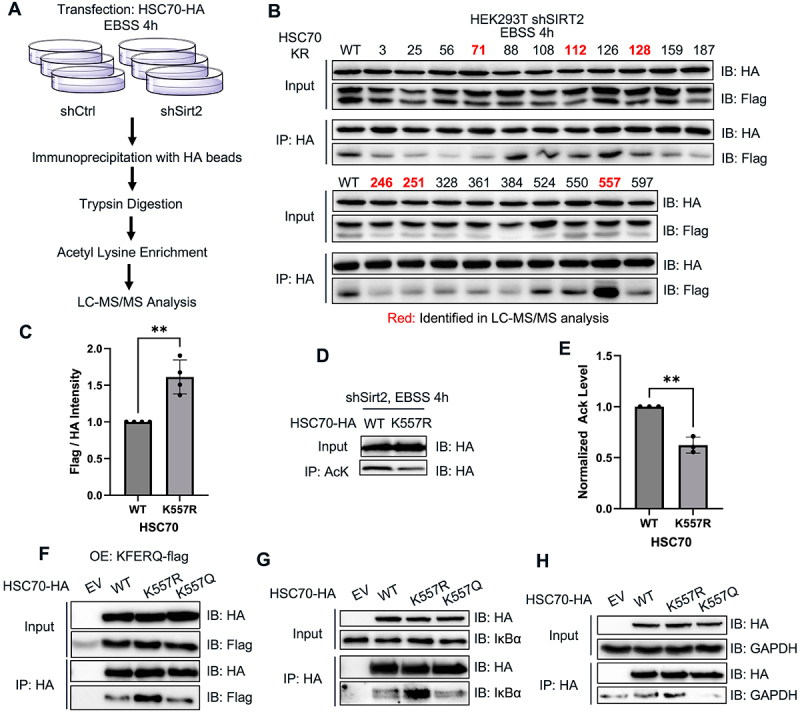
SIRT2-catalyzed deacetylation of K557 regulates HSC70’s CMA substrate binding. (A) Graphic illustration of LC-MS/MS experiment to identify the acetyl-lysine site in HSC70. (B) Interaction between PAmCherry-KFERQ-Flag and various HSC70 mutants. HEK293T cells were transfected with different lysine to arginine (KR) mutants of HSC70-HA and PAmCherry-KFERQ-Flag, and their interaction was analyzed as done in Figure 3B. (C) The HSC70-KFERQ interaction for HSC70 K557R in HEK293T SIRT2KD cells was quantified and normalized against HSC70 WT. (D) Acetylation level of HSC70 WT and K557R in HEK293T shSIRT2 cells detected by western blot. (E) acetylation levels of HSC70 and K557R in (D) was quantified. (F, G, H) the interaction of PAmCherry-KFERQ (F), IκBα (G) and GAPDH (H) with HSC70 WT, K557R and K557Q mutants was observed. Data are means ± SD (*n* = 3). **p < 0.01.

To further confirm and understand the regulatory role of acetylation on HSC70’s function in CMA, we want to pinpoint the specific lysine acetylation sites on HSC70 that affect its binding to CMA substrates. We employed a liquid chromatography-tandem mass spectrometry (LC-MS/MS) approach. HA-tagged HSC70 was overexpressed in HEK293T control and SIRT2-knockdown (SIRT2 KD) cells, followed by a 4-hour starvation treatment with EBSS. After cell lysis, HSC70-HA was purified using HA-affinity beads. The purified HSC70 was then subjected to tryptic digestion, and acetylated peptides were enriched using acetyl-lysine affinity beads. The enriched peptides were subsequently analyzed by LC-MS/MS (Figure 4A) using a data-independent acquisition mass spectrometry mode.

LC-MS/MS analysis identified six lysine residues (K71, K128, K246, K251, K557) whose acetylated peptides were exclusively detected in SIRT2 KD samples (Table 1). To investigate which of these lysine residues are critical for CMA substrate binding, we generated lysine-to-arginine (KR) mutants, which mimic the deacetylated state of the lysine residues. Additionally, anticipating that the proteomics experiments may miss certain acetylation sites, we also created other KR mutants (K3, K25, K56, K88, K112, K126, K159, K187, K328, K361, K384, K524, K550, K597) based on acetylation data of HSC70 from the PhosphoSitePlus database [34].

**Table 1.** List of HSC70 acetyl aysine peptides identified exclusively in shSirt2 samples.

Lysine	Peptide
K71	NQVAMNPTNTVFDAK[Ac]R
K128	MK[Ac]EIAEAYLGK
K137	MKEIAEAYLGK[Ac]TVTNAVVTVPAYFNDSQR
K246	MVNHFIAEFK[Ac]R
K251	K[Ac]DISENK
K557	ATVEDEK[Ac]LQGKINDEDK

Given that SIRT2 promotes HSC70’s binding to CMA substrates, we expected that the HSC70 KR mutant at SIRT2 deacetylation sites would exhibit enhanced binding to CMA substrates compared to HSC70 WT in SIRT2 KD cells. Therefore, we conducted a co-IP screening assay to identify HSC70 KR mutants with significantly increased substrate binding under SIRT2 KD. The assay revealed that the HSC70 K557R mutant exhibited a markedly increased binding affinity for the CMA substrate PAmCherry-KFERQ-Flag (Figure 4B,C), suggesting that HSC70 K557 is the key SIRT2 deacetylation site that regulates CMA substrate binding. To validate whether K557 is a deacetylation site targeted by SIRT2, we compared the acetylation levels of HSC70 WT and HSC70 K557R in HEK293T WT and SIRT2 KD cells. The acetylation level of HSC70 WT was higher than that of HSC70 K557R in SIRT2 KD cells, but not in control shRNA (shCtrl) cells (Figure 4D,E), indicating that K557 is deacetylated by SIRT2. Furthermore, LC-MS/MS analysis identified the acetylated HSC70 K557 peptide exclusively in SIRT2 KD samples (Figure S5). To further validate that K557 acetylation regulates HSC70’s CMA substrate binding, we also generated a lysine-to-glutamine (K557Q) mutant, an acetyl-mimetic mutant, and checked it binding to CMA substrate. In SIRT2 KD cells, the binding of HSC70 to CMA substrate was higher for the K557R mutant than for the WT or K557Q mutant (Figure 4F). The same effect was observed for two other endogenous known CMA substrates, IκBα and GAPDH (Figure 4G,H). Collectively, these data suggest that acetylation of HSC70 K557 plays an important role in regulating its binding ability to CMA substrates.

### Deacetylation of HSC70 K557 modulates CMA activity

**Figure 5.**
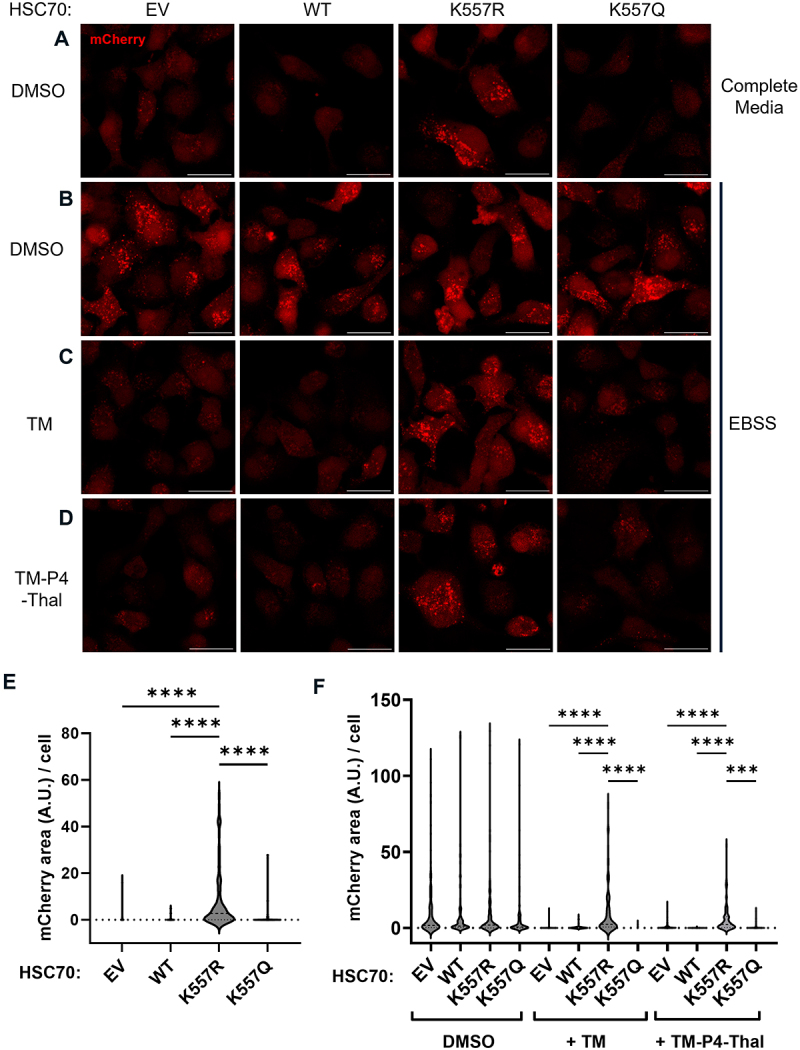
Deacetylation of HSC70 K557 modulates CMA activity. (a) A547 mCherry-KFERQ cells overexpressing empty vector (EV), HSC70 WT, K557R, or K557Q were observed under nutrient rich conditions. (B-D) A547 mCherry-KFERQ cells overexpressing EV, HSC70 WT, K557R or K557Q were treated with DMSO control (B), TM (C), or TM-P4-Thal (D) and observed under nutrient-deprived conditions. (E) quantification of data in (A). (F) quantification of data in (B-D). Data are means ± sd. ** *p* < 0.01. Scale bar = 20 μm.

To investigate whether the acetylation of K557 affects CMA activity, we analyzed CMA function in cells overexpressing various HSC70 mutants. We observed a modest increase in constitutive CMA activity under nutrient-rich conditions (using complete media) in cells expressing HSC70 K557R, but not in cells overexpressing EV control, HSC70 WT, or HSC70 K557Q (Figure 5A,E). This suggested that overexpression of HSC70 K557R alone is sufficient to activate CMA in the cells.

Next, we wanted to see whether the overexpression of K557R could rescue the lack of SIRT2. When the cells were stressed with nutrient deprivation with EBSS for 4 h, we observed a significant increase in CMA activity (Figure 5B,F). When the cells were treated with SIRT2 inhibitor or degrader during nutrient stress, we observed that only the cells overexpressed with HSC70 K557R rescued the downregulation of CMA activity caused by SIRT2 inhibition or degradation, while HSC70 WT, K557Q did not (Figure 5C,D,F). The data further support that deacetylation of HSC70 at K557 promotes CMA activation in the cells.

A recent report [35] suggested that two inhibitors that mimic calorie restriction through depletion of cellular acetyl-CoA or inhibition of acetyltransferases can promote CMA by unknown mechanisms. To test if HSC70 acetylation is involved in this process, we followed the protocol described in the previous paper and treated KFERQ-PAmCherry CMA reporter cell line with C646 (p300 inhibitor) or SB204990 (ACLY inhibitor) for 48 hrs. At the meantime, we overexpressed either HSC70 WT or HSC70 K557Q to see if the acetylation mimic can rescue the CMA induced by cellular acetyl-CoA depletion. The result suggested that HSC70 K557Q can indeed rescue the CMA induced by C646 or SB204990 (Figure S6). Thus, our finding on the role of HSC70 K557 acetylation in CMA provides an explanation to the previously reported effect of acetyl-CoA depletion or acetyltransferases inhibition on CMA.

## Discussion

**Figure 6.**
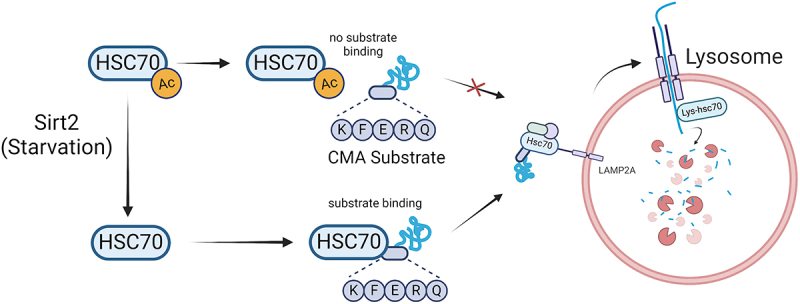
Schematic illustration for HSC70 deacetylation-induced chaperone-mediated autophagy. During nutrient rich conditions, HSC70 is acetylated and there is no substrate binding to chaperone-mediated autophagy (CMA) substrates with KFERQ-like motif, so CMA is not activated. During starvation conditions, SIRT2 is activated and deacetylates HSC70 at K557. Deacetylated HSC70 binds strongly to KFERQ-like substrates, which promotes CMA. Figured created using BioRender.

In this study, we uncover a regulatory mechanism by which SIRT2-mediated deacetylation of HSC70 enhances chaperone-mediated autophagy (CMA) activity under nutrient deprivation. Specifically, we demonstrate that SIRT2 directly interacts with and deacetylates HSC70 at K557, which is critical for HSC70’s binding to CMA substrate proteins with the KFERQ-like motif during nutrient stress conditions (Figure 6). These findings provide important insights into the role of acetylation in modulating the activity of HSP70 family proteins and highlight the interplay between protein post-translational modifications and autophagic pathways in cellular stress responses.

One of the most intriguing findings of this study is the identification of K557 on HSC70 as a key deacetylation site required for CMA substrate binding. Acetylation at this residue impairs HSC70’s ability to interact with KFERQ-like motifs, while its deacetylation enhances substrate affinity. This serves as yet another example that highlights the importance of acetylation as a regulatory mechanism for HSP70 family proteins in stress responses [22,23]. Notably, our results show that overexpression of a deacetylation-mimetic mutant (HSC70 K557R) enhances CMA activity even under nutrient-rich conditions, further supporting the functional significance of K557 deacetylation in regulating CMA.

Our LC-MS/MS acetylation site analysis revealed multiple potential residues on HSC70 regulated by SIRT2, but K557 stood out as the critical site for CMA modulation. While other lysine residues may also be deacetylated by SIRT2, their roles appear less significant in the context of CMA substrate binding, as evidenced by the lack of substrate-binding enhancement in mutants other than K557R. This specificity highlights K557 as a key site for CMA substrate binding.

In recent years, using small molecules to target specific proteins for degradation has become very popular. Accordingly, small molecules that target proteins for CMA degradation have also been reported, which utilize structures that mimic the “KFERQ” motif [36–38]. While HSC70 is well documented to bind to the “KFERQ”-motif containing CMA substrates, the binding mode and even the binding site on HSC70 for this motif is not known. The finding that K557 acetylation is important for the binding of the “KFERQ”-motif suggests that regions near K557 are likely involved in binding the CMA substrate. This knowledge may help future efforts in designing CMA-targeting protein degraders.

While CMA is known to be activated by nutrient deprivations, the molecular mechanism underlying this is not well known. Our study provides a molecular mechanism via which amino acid starvation activates CMA. Our data suggest that amino acid starvation induces SIRT2 activation, facilitating its deacetylation of HSC70 to enhance CMA activity by increasing HSC70’s affinity to CMA substrates. This mechanism potentially serves as an early phase adaptive response to nutrient deprivation in addition to other CMA adaptive responses such as increased levels of LAMP2A [39,40], allowing cells to efficiently clear unnecessary proteins and recycle amino acids for essential processes during the stress conditions.

Additionally, our finding on the role of HSC70 K557 acetylation in CMA provides an explanation to the previously reported effect of acetyl-CoA depletion or acetyltransferases inhibition on CMA [35]. The acetyl-CoA depletion or acetyltransferase inhibition would decrease HSC70 K557 acetylation and thus promote CMA. Interestingly, CMA itself also degrades enzymes that produce acetyl-CoA [41,42]. Thus, there is a possibility that a positive feedback loop exists that further promotes CMA by decreasing acetyl-CoA and HSC70 acetylation level. Along this line, it is also worth noting that SIRT2 is known to deacetylate and inhibit ACLY and ACSS2 [43,44], two enzymes that produce acetyl-CoA. The regulation of SIRT2 on HSC70, ACLY, and ACSS2 could thus all contribute to the promotion of CMA under amino acid limitation. As we showed that amino acid starvation induces SIRT2 activation, these findings put SIRT2 as a central hub regulating many different proteins to coordinate the stress response to amino acid limitation. Future studies will be needed to further establish this model.

Our findings underscore the intricate regulation of autophagy by protein post-translational modifications, particularly acetylation and deacetylation. CMA has been connected to several human diseases, including neurodegeneration, cancer, and aging [35,45,46]. By linking SIRT2 activity to CMA regulation, our study not only advances our understanding of autophagic pathways but also raises the possibility of targeting SIRT2-HSC70 interactions as a therapeutic strategy for diseases associated with CMA dysregulation, such as neurodegeneration, cancer, and aging [14,15].

## Methods and materials

### Reagents, antibodies, and plasmids

pCMV3-HSC70-HA (#HG11329-CY) and pCMV3-SIRT2-HA (HG10830-CH) plasmid were obtained from SinoBiological. pSIN-PAmCherry-KFERQ-NE was a gift from Shu Leong Ho (Addgene plasmid # 102,365; http://n2t.net/addgene:102365; RRID:Addgene_102365). Primary Antibodies from Invitrogen: HSC70 (MA1-26078); Cell Signaling Technologies: SIRT2 (#12650), Santa Cruz Biotechnology: HA-HRP (sc-7392); Flag-horse radish peroxidase (Sigma #A8592) was used to immunoblot for Flag-conjugated proteins at 1:5000 dilution. Precision Plus Protein™ All Blue Prestained Protein Standards (Biorad #1610393) was used for molecular size markers for immunoblotting (Western blot).

### Stable cell line generation

SIRT2 shRNA and PAmCherry-KFERQ-NE lentiviral particles were generated using HEK293T cells by co-transfection of SIRT2 shRNA pLKO.1-shSIRT2 (Sigma cat# TRCN0000040219) or pSIN-PAmCherry-KFERQ-NE (Addgene #102365) plasmid with coating and packaging vectors. The virus-containing media was then harvested and A549 cells were transduced with the lentiviral particles. After lentiviral transduction, the cells were then selected using puromycin for 2 days.

### Generation of HSC70-HA mutants

The PCR and mutagenesis were performed using the New England Biolabs Q5® Site-Directed Mutagenesis Kit according to the manufacturer’s instructions. Primers TGAAGATGAGcgcCTTCAAGGCAAGATTAAC and ACAGTTGCTTTCATGTTG were used for K557R, and TGAAGATGAGcagCTTCAAGGCAAG and ACAGTTGCTTTCATGTTG were used for K557Q mutant. The mutations were confirmed by Sanger sequencing by Genewiz.

### Binding assay of HSC70 to CMA substrates

pSIN-PAmCherry-KFERQ-NE was converted to PAmCherry-KFERQ-flag using primers gatgatgataaaTAAGTTTAAACAATCACTAGTTCG and atctttataatcGCTGGGGGAACTGTC. The PAmCherry-KFERQ-Flag and HSC70-HA (WT or mutant) were co-overexpressed using PEI transfection. The cells were lysed with RIPA lysis buffer with a protease inhibitor cocktail, and HSC70-HA was pulled down using anti-HA magnetic beads (Thermo #88836). The pulled-down proteins were eluted, and levels of HSC70-HA and PAmCherry-KFERQ-flag were observed using immunoblotting and quantified using ImageJ.

### Imaging procedures of CMA reporter

Cells stably overexpressing PAmCherry-KFERQ-NE were seeded onto a poly-D-lysine coated 35 mm dish with a 14 mm glass diameter coverslip (Mattek #P35GC-1.5–14-C) on the bottom and incubated overnight. Photoactivation of cells grown on coverslips was carried out with a 395 nm LED light (uvBeast V3) for 5 min using 8100 uW cm^−2^ light intensity. Then cells were treated with EBSS media for 4 hours. The cells were then fixed with 4% paraformaldehyde/PBS for 10 minutes and washed with PBS 3 times. The cells were then placed on the fluorescent microscope to observe the mCherry signal.

### Transfection of plasmids

HEK293T cells were plated in a 6-well plate 24 h prior to the transfection. Plasmids were transfected using polyethyleneimine hydrochloride (PEI, Polysciences) at a ratio of 1:3 for DNA:PEI. A549 cells were transfected by Lipofectamine 3000 (Invitrogen) following the manufacturer’s instructions.

### Sample preparation for liquid chromatography and mass spectrometry

HEK293T shControl and shSIRT2 were grown in a 15-cm plate and transfected with HSC70-HA. HSC70-HA was purified using anti-HA magnetic beads (Thermo #88836). The proteins were eluted using 1% SDS in PBS. Eluted proteins were then subjected to reduction, alkylation, and trypsin digestion using the S-Trap™ proteomics sample preparation kit (Protifi). The resulting peptides were then enriched with acetyl-lysine affinity beads to increase the signal (Cytoskeleton, AAC04) and eluted with 0.15% trifluoroacetic acid (TFA) in HPLC grade water. The peptides were lyophilized and subjected to LC-MS/MS analysis.

## Liquid chromatography and mass spectrometry

The tryptic digests of samples were reconstituted in with buffer A (0.1% formic acid in water) for nanoLC-MS/MS analysis. The analysis was carried out using a timsTOF HT (Bruker Daltonics, Bremen, Germany) mass spectrometer equipped with a CaptiveSpray nanoelectrospray ion source, and coupled with a nanoElute2 system (Bruker Daltonics, Bremen, Germany). Peptide samples were injected onto an Ion Opticks column (1.5 µm, 150 µm i.d. x 250 mm) at 250 nL/min flow rate at 50°C. The peptides were eluted via a gradient of 2% to 23% buffer B (0.1% formic acid in acetonitrile) over 18 min, 23 to 35% B over 4 min, followed by a 4 min ramping to 90% B and a 4 min hold at 90% B. The separated peptides were ionized by a CaptiveSpray ion source, introduced into the timsTOF HT and analyzed by ddaPASEF. The singly charged precursor ions were excluded with a polygon filter. The capillary voltage was set to 1600 V, and the dry gas was at 3.0 L/min with a dry temperature of 180°C. Five PASEF ramps were acquired with a ramp time set to 100 ms with 100% duty cycle, the mass range was set as 100–1700 m/z and the ion mobility range was 0.60–1.50 1/K0. The collision-induced dissociation energies were linearly ramped as a function of ion mobility ranging from 20 (1/K0 = 0.6 Vs cm^−2^) to 59 eV (1/K0 = 1.6 Vs cm^−2^)

### Statistics

GraphPad Prism software was used for statistical data analysis.

## Supplementary Material

Sirt2_HSC70 manuscript_20250624_Autophagy Reports_Supplement.docx

## Data Availability

The data that support the findings of this study are available from the corresponding author upon reasonable request.

## Abbreviations

CMA: chaperone-mediated autophagy
co-IP: co-immunoprecipitation
CRISPR: clustered regularly interspaced short palindromic repeats
EBSS: Earle’s Balanced Salt Solution
HEK293T: Human embryonic kidney 293T cells
HSC70: heat shock cognate 71 kDa protein
HSP70: heat shock protein 70, HSP: heat shock protein
KFERQ-motif: Lysine (K) Phenylalanine (F) Glutamic acid (E) Arginine (R) and Glutamine (Q) motif
KQ: lysine-to-glutamine mutant
KR: lysine-to-arginine mutant
LAMP2A: lysosome-associated protein type 2A
LC-MS/MS: liquid chromatography-tandem mass spectrometry
PAmCherry: photoactivable mCherry
PTM: post-translational modification
SIRT2: Sirtuin 2
SIRT2KD: Sirt2 knockdown
TM: thiomyristoyl
WT: wildtype
