# Supplementary material for: Deacetylation of HSC70 by SIRT2 promotes chaperone mediated autophagy
Source: Autophagy Rep. 2025 Nov 12;4(1):2580781. doi: 10.1080/27694127.2025.2580781 (PMC12622308; doi:10.1080/27694127.2025.2580781)
Supplement: Sirt2_HSC70 manuscript_20250624_Autophagy Reports_Supplement.docx [file KAUO_A_2580781_SM3057.docx]

**SUPPLEMENTAL INFORMATION**

**Deacetylation of HSC70 by SIRT2 Promotes Chaperone Mediated Autophagy**

Byunghyun Ahn^a,b,c,=^, Wenzhe Chen^a,h,=^, Wenbiao Shi^a,d,e^, Ruben Shrestha^f^, Fenghua Hu^b,c^, and Hening Lin^a,b,g,h,*^

^a^ Department of Chemistry and Chemical Biology, Cornell University, Ithaca, NY 14853, United States

^b^ Department of Molecular Biology and Genetics, Cornell University, Ithaca, NY 14853, United States

^c^ Weill Institute for Cell and Molecular Biology, Cornell University, Ithaca, NY 14853, United States

^d^ Department of Nutrition and Health, China Agricultural University, Beijing 100091, China

^e^ Key Laboratory of Precision Nutrition and Food Quality, Beijing 100091, China

^f^ Bruker Daltonics, San Jose, CA 95134, United States

^g^ Howard Hughes Medical Institute, Cornell University, Ithaca, NY 14853, United States

^h^ Howard Hughes Medical Institute, Department of Medicine and Department of Chemistry, University of Chicago, Chicago, IL 60637, United States

* Corresponding author: Hening Lin

^=^ Authors have contributed equally

Corresponding author email: linh1@uchicago.edu

**
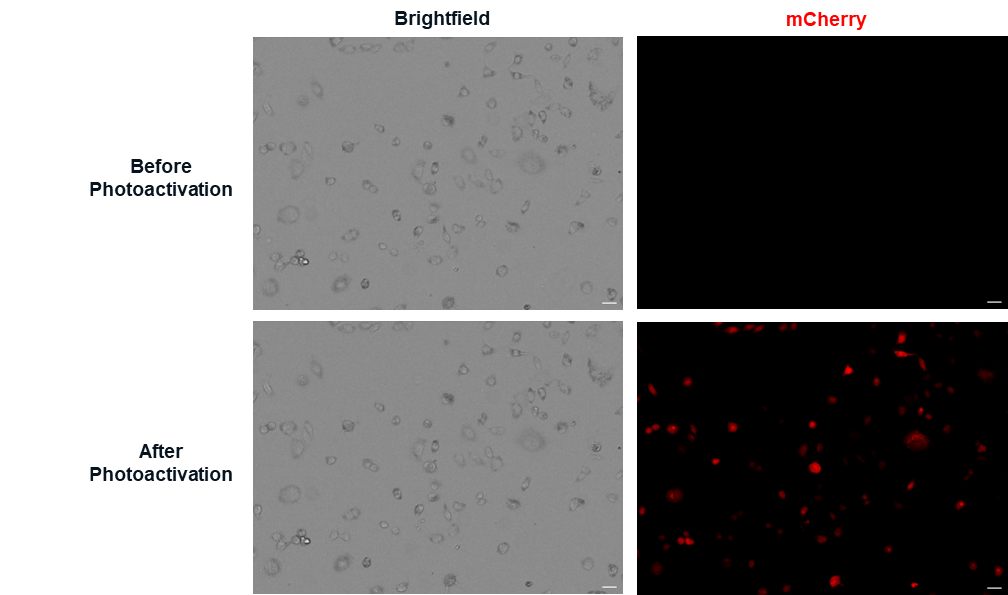
**

**Figure S1. Verification of A549 KFERQ-PAmChery cell line generation.** A549 cells overexpressing KFERQ-PAmCherry was observed before and after photoactivation with UV light.


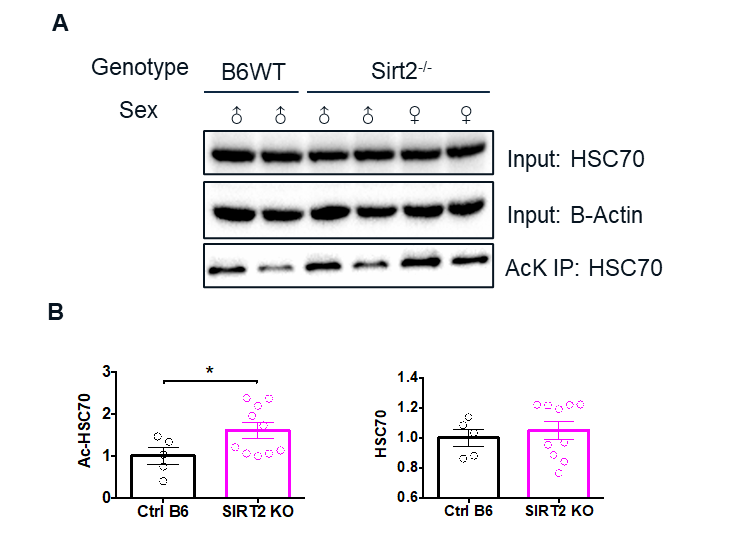


**Figure S2. HSC70 is acetylated in SIRT2KO mice brain samples.** (A) Brain samples were collected from C57BL/6 wildtype and SIRT2^-/-^ mice, and HSC70 acetylation level was observed. (B) Quantification of data replicates of (A). Data are means ± SD. * P < 0.05.


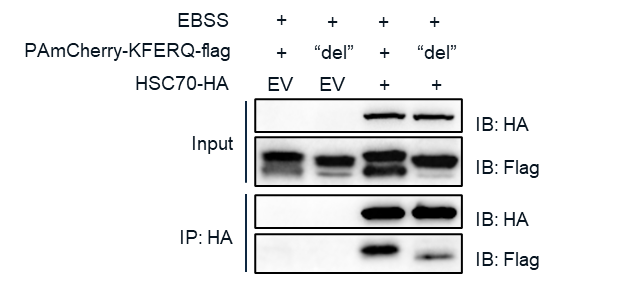


**Figure S3.** **KFERQ motif is important for HSC70 binding.** PAmCherry-KFERQ-flag was cloned so that the “KFERQ” motif was deleted in from the sequence (“del”), and its interaction with HSC70 was observed.

HA / HSC70

Sirt2

HSC70-HA

-

-

TM

TMP4

Sirt2

IP: HA

-

+

+

+

IκBα

GAPDH

-

-

TM

TMP4

-

+

+

+


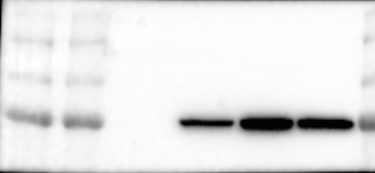

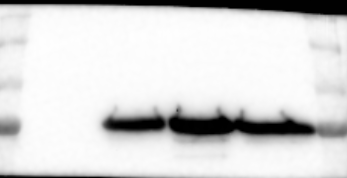

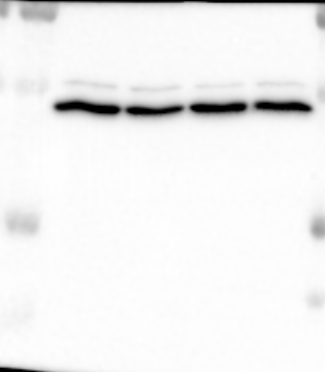

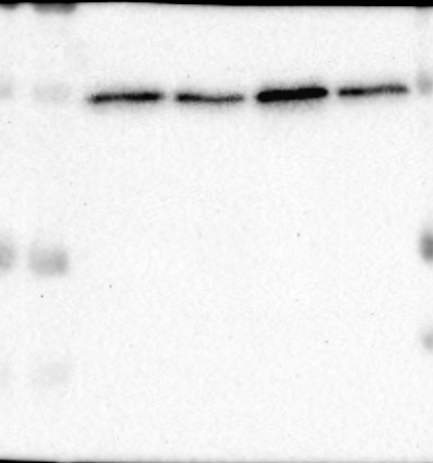

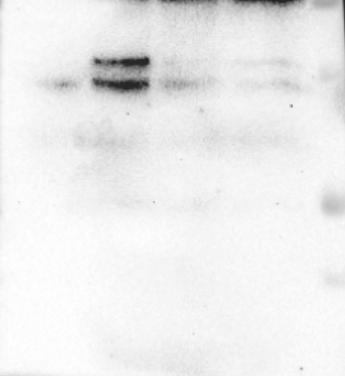

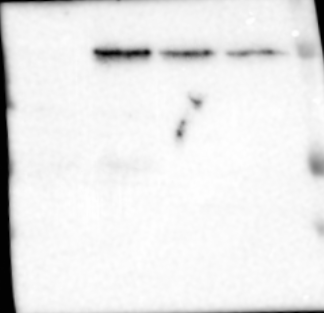

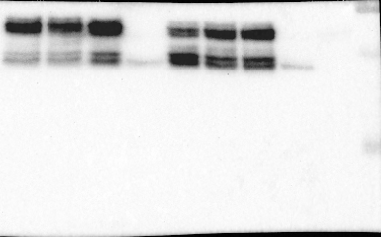


**Figure S4. SIRT2 inhibition disrupts HSC70 binding with its endogenous CMA substrates.** HEK293T cells were transfected with HSC70-HA, then starved and treated with SIRT2 inhibitors TM or TM-P4-Thal (TMP). HSC70 binding to two endogenous CMA substrates (IκBα and GAPDH) was observed using co-IP and Western blot.


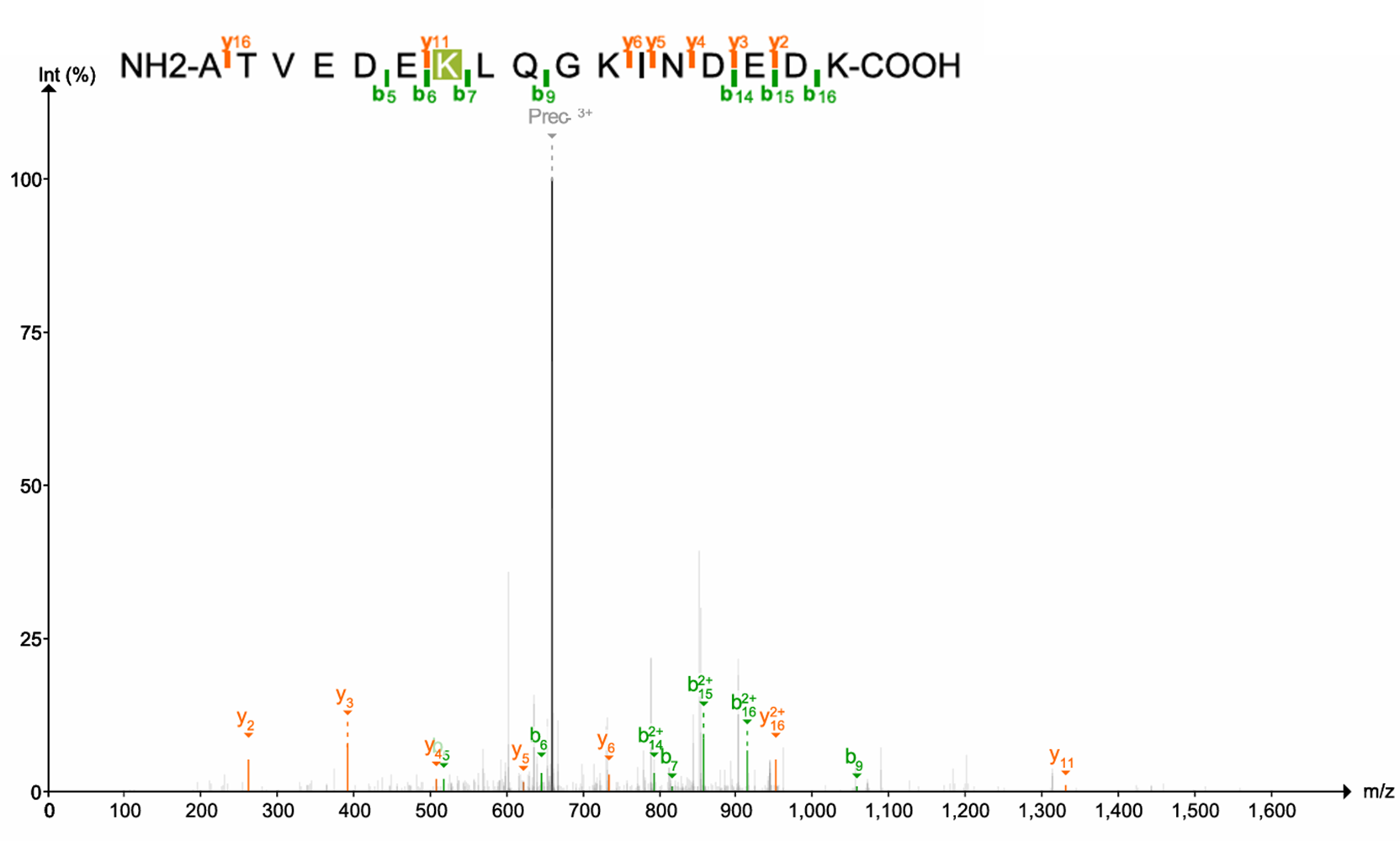


**Figure S5. Fragmentation peaks of HSC70 K557Ac peptide ATVEDEK[Ac]LQGKINDEDK from LC-MS/MS analysis in SIRT2KD samples.**

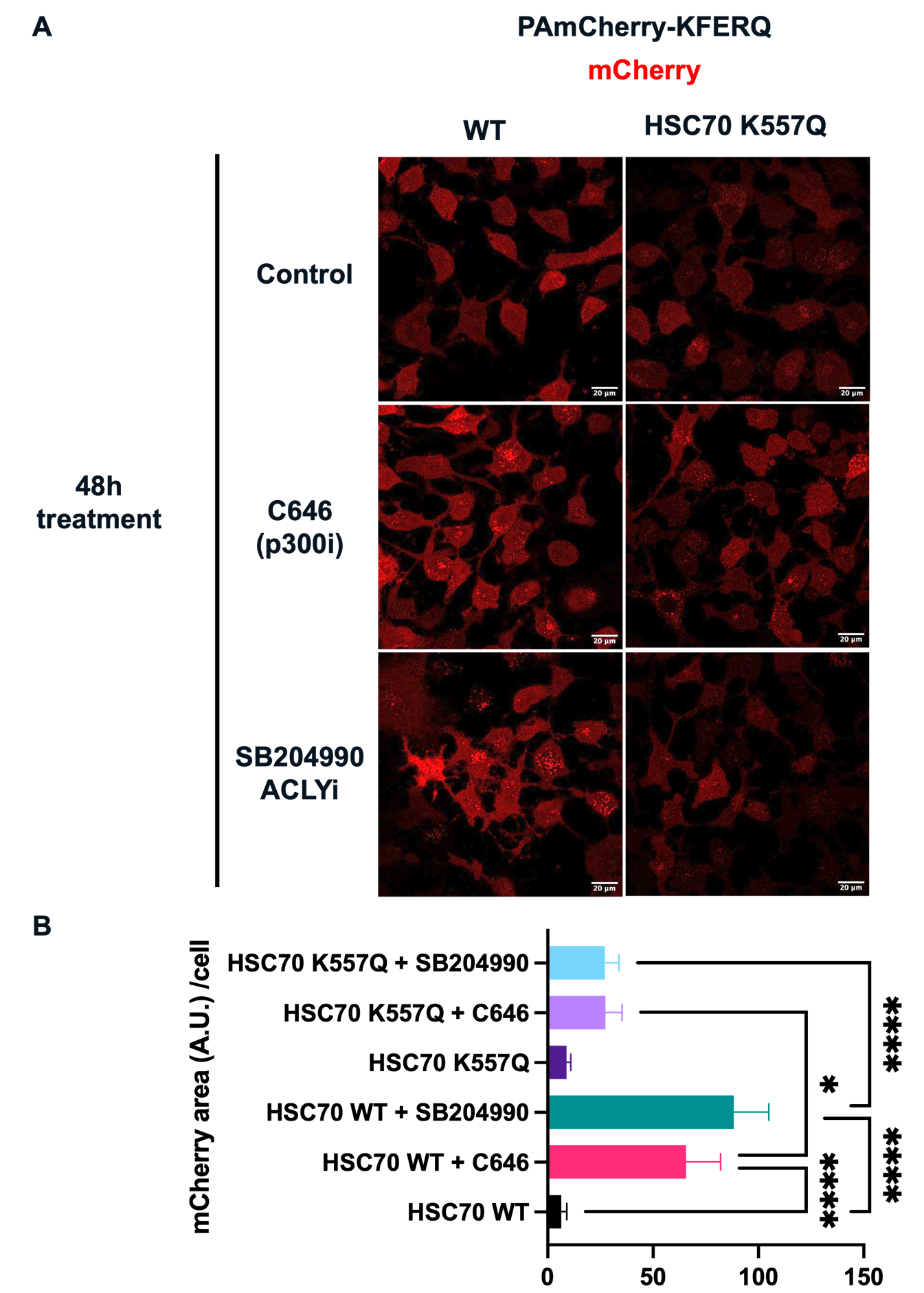


**Figure S6. HSC770 K557Q Acetylation mimic can rescue the increase in CMA caused by acetyl-CoA depletion**. (A) A547 mCherry-KFERQ cells overexpressing HSC70 WT or K557Q was treated with either DMSO, C646 (10uM) or SB204990 (50uM) for 48 hrs. The CMA level was observed by the formation of puncta under microscope and quantified in (B). Data are means ± SEM. ** P < 0.01. Scale bar = 20μm.
